# Acupuncture at Zusanli (ST36) for Experimental Sepsis: A Systematic Review

**DOI:** 10.1155/2020/3620741

**Published:** 2020-03-07

**Authors:** Fang Lai, Yang Ren, Chengzhi Lai, Rui Chen, Xuelian Yin, Caixia Tan, Jiansen Li, Chunmei Yang, Guorong Liang, Jun Li, Ruifeng Zeng

**Affiliations:** ^1^The Second Clinical College of Guangzhou University of Chinese Medicine, Guangzhou 510006, Guangdong, China; ^2^The Second Affiliated Hospital of Guangzhou University of Chinese Medicine, Guangdong Provincial Hospital of Chinese Medicine, Guangdong Provincial Key Laboratory of Research on Emergency in TCM, Guangzhou 510120, Guangdong, China; ^3^Chao En-Xiang Famous Chinese Medicine Expert Inheritance Studio, Guangdong Provincial Hospital of Chinese Medicine, Guangzhou, Guangdong, China; ^4^Bao'an Traditional Chinese Medicine Hospital, Guangzhou 510120, Guangdong, China; ^5^Mianyang Hospital of T.C.M., Mianyang 621000, Sichuan, China; ^6^The Clinical Medical College of Acupuncture Moxibustion and Rehabilitation of Guangzhou University of Chinese Medicine, Guangzhou 510006, Guangdong, China

## Abstract

**Background:**

Sepsis is a global major health problem with high mortality rates. More effective therapy is needed for treating sepsis. Acupuncture has been used for various diseases, including severe infection, in China for more than 2,000 years. Previous studies reported that acupuncture at Zusanli (ST36) might be effective in treating sepsis, but the efficacy and the quality of evidence remain unclear since there is no systematic review on acupuncture at ST36 for sepsis.

**Methods:**

Seven databases were searched from the inception of each database up to May 2019. Ultimately, 54 studies using acupuncture at ST36 for the treatment of experimental sepsis were identified in both English and Chinese literature with systematic review procedures.

**Results:**

Acupuncture might be useful in reducing injuries induced by sepsis in cardiac, lung, kidney, liver, gastrointestinal tract, and immune system. Its potential mechanisms for antisepsis might include reducing oxidative stress and inflammation, improving microcirculatory disturbance, and maintaining the immune balance mediated by dopamine. However, the positive findings should be interpreted with caution due to poor methodological quality and publication bias.

**Conclusion:**

Acupuncture at ST36 might be a promising complementary strategy for controlling sepsis inflammation, yet further studies are needed.

## 1. Introduction

Sepsis is a systemic clinical syndrome induced by inflammatory response from severe infections. The overwhelming inflammatory responses to sepsis may cause multiple organ failure as a result [1–4], which makes sepsis the leading cause of mortality in noncoronary Intensive Care Units in the world nowadays [5, 6]. Although the guidelines of “Surviving Sepsis Campaign” have led to great advances in sepsis management [7] and new antibiotics might be temporarily efficient in helping infection control, the mortality rates are still high and more effective therapy is still urgently needed [8, 9].

Acupuncture is one important therapeutic method in traditional Chinese medicine (TCM). It involves the insertion of fine needles in defined points, which is named “acupoints,” and usually is followed by stimulation of manual techniques or electrical devices [10]. It has been reported that acupuncture may have a bidirectional regulating effect and antagonize systemic inflammatory response [11–14]. Zusanli (stomach meridian, ST36) is an acupoint located at 3 cm below the knee joint on the anterior aspect of the leg according to the TCM theory of acupuncture. In the past decade, studies reported the potential of acupuncture at ST36 for infectious diseases due to its numerous effects, such as anti-inflammatory [15], immunoenhancing [16, 17], antioxidative [18], accelerating the recovery of various gastrointestinal disorders [19]. Recent studies have further researched acupuncture at ST36 as a treatment for sepsis in humans and animal models [20, 21]. However, there is no systematic review of acupuncture at ST36 in treating sepsis; thus systematic assessment of its efficacy and mechanisms is still lacking. Furthermore, systematic reviews of preclinical animal data may help predicting the magnitude and direction of therapeutic effects in human trials [22], identifying potential candidates worthy of further basic research, precluding unnecessary study replication, and contributing to refinement in animal experimentation [23, 24]. Herein, we report a systematic review of acupuncture at ST36 in experimental sepsis in this paper with the following objectives:

1. systematically review and evaluate the experimental evidence for acupuncture at ST36, either before or after the onset of sepsis in animal models;
2. determine the efficacy of acupuncture at ST36 in sepsis and explore the impact on the efficacy;
3. analyze the possible mechanisms of acupuncture at ST36 in treating sepsis;
4. propose the refinement for design of future experimental studies and ultimately further clinical trials in human patients in sepsis.

## 2. Methods

### 2.1. Search Strategy

We identified studies of acupuncture at ST36 in sepsis animal models from PubMed, the Cochrane Central Register of Controlled Trials (CENTRAL) in The Cochrane Library, EMBASE, Chinese National Knowledge Infrastructure (CNKI), VIP Database, Wanfang Data, and Chinese Biomedical Literature Database (CBM) by using the terms “ST36” OR “zusanli” OR “Tzusanli” OR “Electro-acupuncture” OR “Electroacupuncture” OR “EA” OR “acupuncture” OR “acupuncture electric stimulation” OR “AES” AND “sepsis” OR “septicemia” OR “septic shock” OR “endotoxic shock” OR “toxic shock” OR “bloodstream infection” OR “blood stream infection” in English or in Chinese, when appropriate, without language restrictions (search terms for PubMed are listed in the appendix). All databases were searched from the inception up to May 2019. All searches were limited to studies on animals. All included articles and relevant reviews were also hand-searched for the reference lists. Two authors (Rui Chen and Chunmei Yang) identified studies from databases independently. Disagreements were solved after discussion with a third party (Fang Lai).

### 2.2. Eligibility

Studies must be concerning the effectiveness of acupuncture at ST36 for sepsis, regardless of method of acupuncture (electroacupuncture (EA) or manual acupuncture (MA)), EA waveform parameters, EA current density, EA pulse width, frequency, or course of treatment. To prevent bias, we prespecified the inclusion criteria as follows: (1) studies were included when comparing acupuncture at ST36 as monotherapy or an adjuvant therapy for experimental sepsis and (2) effectiveness was compared with a control group receiving the sham acupuncture therapy, no treatment, positive drug therapy, or the same supporting treatment (such as fluid resuscitation and antibiotics) as the treatment group. Exclusion criteria were preset as follows: (1) studies comparing acupuncture at ST36 as a monotherapy to Chinese herbal medicine (CHM) or acupuncture at another acupoint; (2) duplicate publications; and (3) not focusing on experimental sepsis. Two authors (Guorong Liang and Jiansen Li) screened for included studies independently. Discussions with a third party (Fang Lai) were carried out to solve disagreements.

### 2.3. Data Extraction

We extracted details from each study as follows: (1) the first author's name and the publication year; (2) model of sepsis, model induction method details, anesthetized method, number of animals, animal species, sex, age, weight, comorbidities (such as hypertension, diabetes, and aged), and study design; (3) method of acupuncture, waveform parameters, current density, frequency, pulse width, course of treatment, intervention of control group, and supportive care for animals; and (4) outcome measures, outcomes assessments time, and intergroup differences.

If the data were missing or conflicting, we tried to contact the authors to get additional information. When no further information is available and critical data remain missing or conflicting, the study would be excluded. Two authors (Xuelian Yin and Caixia Tan) extracted data independently. Discussions were carried out with a third party (Fang Lai) to solve any disagreements.

### 2.4. Quality Assessment

We utilized a modified scale to evaluate the methodological quality of the included studies [25–28]: (1) peer-reviewed publication; (2) control of temperature, humidity, and light; (3) randomized allocation; (4) reporting details of randomized allocation method; (5) allocation concealment; (6) blinded model induction; (7) blinded intervention administration; (8) blinded outcome assessment; (9) sample size calculation or explanation; (10) animal welfare regulations compliance statement; (11) being free of selective reporting; (12) potential conflict of interests statement; (13) reporting statistical method; (14) reporting numerical data in Results; (15) reporting actual numbers of animal samples of different groups in Results; (16) completeness of follow-up; and (17) intention-to-treat analysis. A “yes” for the item attributed one point and was denoted by a “+,” a “partially yes” attributed 0.5 points with a “±,” an “unclear” attributed 0 points with a “?,” and a “no” attributed 0 points with a “–.” Two authors (Yan Ren and Chengzhi Lai) assessed study quality independently. Disagreements were solved after discussion with a third party (Jun Li).

## 3. Results

### 3.1. Study Selection

**Figure 1:**
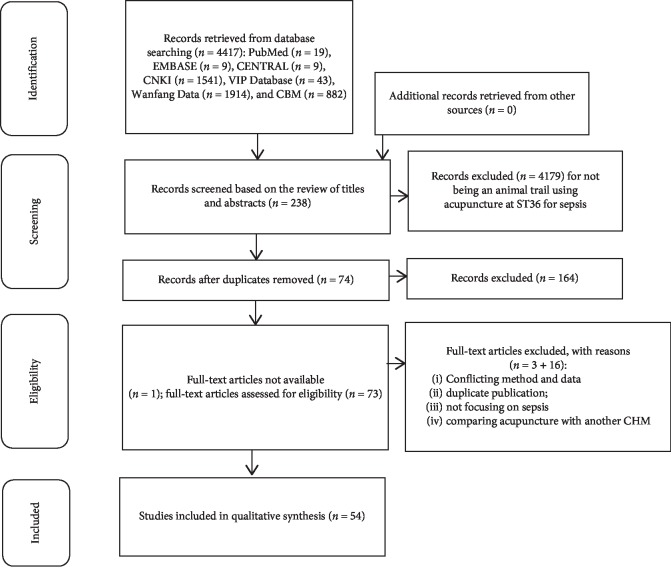
Flow diagram showing study selection process. CENTRAL, the Cochrane Central Register of Controlled Trials in The Cochrane Library; CNKI, Chinese National Knowledge Infrastructure; CBM, Chinese Biomedical Literature Database; CHM, Chinese herbal medicine.

4417 papers were retrieved from the seven above-mentioned databases. 4179 papers were excluded with at least one of the following reasons: (1) being a case report or review; (2) not being an animal research; and (3) not focusing on sepsis. 238 articles remained after going through the titles and abstracts, of which 164 records were removed for being duplicates. Another 1 paper was excluded for no full-text article was available with effort. By full-text reviewing, 16 studies were excluded according to the exclusion criteria, and another 3 articles were excluded for the contradiction between the method and the result. Ultimately, 54 eligible studies were identified. Summary of the study selection process was listed in a flow diagram (Figure 1) [21, 29–81].

### 3.2. Study Characteristics

#### 3.2.1. Time and Place of Studies

The 54 included trials were published or written (if it was an unpublished thesis) between 2006 and 2018. 17 of the tests (about 31%) were within the last five years. All conducted in China.

#### 3.2.2. Experimental Animals

The animal species included Sprague-Dawley (SD) rats, Wistar rats (including 3 articles [54, 55]which reported as “Wastar rats” by mistake), Wild-type C57BL/6J mice, and New Zealand white rabbits (including 3 articles [32, 39, 46] reported as “New England rabbits” by mistake).

96.3% of the trials utilized male animals (52 out of the 54 trials), while only 1 trial [62] used both male and female animals and 1 study [49] did not report the sex of the animals.

Only 6 studies [51, 52, 61, 64, 75, 77] reported using adult animals, 16 studies [30, 34, 38, 40, 49, 50, 53, 57, 62, 65, 68, 69, 73, 78–80] had no statement about animals' age, and the remaining 33 studies reported the specific age of the animals.

Eight of the included articles [61, 63, 65, 67, 70, 71, 75, 76] utilized specific pathogen-free (SPF) animals, 3 articles [50, 58, 62] reported that all animals utilized were healthy, 18 articles [29–31, 33–38, 40, 42–45, 47, 48, 64, 72] had statement as using “healthy and clean” animals, and another 7 articles [49, 53, 57, 60, 68, 78, 81] reported using “clean animals,” while 19 articles did not report whether there were any comorbidities in animals.

#### 3.2.3. Sepsis Models

27 of the 54 studies (50.0%) were lipopolysaccharide (LPS) injection model, the dosage of LPS varied from 5 mg/kg to 30 mg/kg in rodents and was 5 mg/kg for rabbits, 3 studies [21, 51, 52] administrated LPS through intraperitoneal (IP) injection, and 24 studies [29–49, 53, 63, 68] administrated them through intravenous (IV) injection.

48.1% (26 out of 54) [21, 50, 54–62, 64–66, 69, 72–81] of the included studies utilized cecal ligation and puncture (CLP) model. The length of the ligated cecum varied from 50% to the whole length of the cecum. Only several studies reported the type of suture used for ligation (no. 0 [72, 74, 77] or no. 30 silk [57, 58, 62], 3 of the 26 reports, respectively), the number of puncture administration (one-time [21, 57, 58, 62, 74, 77, 80] or three times [54, 55, 59–61, 64, 79], 7 of the 26 reports, respectively), the size of needles used for puncture (no. 16 needles in 10 studies [54, 55, 59–61, 64, 66, 76, 79, 81] out of the 26 reports) and the number of puncture pores (two pores in 7 studies [50, 57, 58, 62, 72, 74, 77] out of 26 studies). Moreover, there were some studies not reporting the length of the ligated cecum [57, 58, 62], times of perforation administration [50, 72, 78], or any details of the CLP [56, 65, 75].

One study [21] utilized both LPS injection model and CLP model in different episodes, while 3 studies reported utilizing the 20–35% of the total body surface area (TBSA) III degree burned on the back of the rat by boiling water (12–15 s), following by a 5 mg/kg Muramyl dipeptide IV injection [67, 70, 71].

Urethane [30, 31, 34–38, 40, 42–45, 47–50] was used in 16 studies to induce anesthesia, pentobarbital [41, 63, 65, 66, 72–74, 76, 77, 80, 81] was used in 11 studies, ketamine [32, 57, 58, 62] was used in 4 studies, chloral hydrate [29, 78] was used in 2 studies, and xylazine hydrochloride [33] and isoflurane [64] were used in 1 study, respectively. Ketamine combined with Suxinmian [54–56, 59–61, 79], midazolam [67, 70, 71], xylazine [21, 69], and urethane [39, 46] were used in 7, 3, 2, and 2 studies, respectively. While the remaining 5 studies [51–53, 68, 75] had no statement about anesthetics.

Details about sepsis models of the included studies are summarized in Table 1.

#### 3.2.4. Sample Sizes

50 articles reported the design number of animal in the treatment group (TG) for each outcome index at each time phase, which varied from 3 to 20, while 4 articles [32, 41, 49, 69] did not report the design number of animals in each subgroup. 44 articles reported the actual numbers of animal samples of different groups in Results, 1 article [30] partially reported, while 9 articles [32, 51–53, 58, 59, 68, 78, 81] had no report on numbers of samples in Results.

#### 3.2.5. Acupuncture Intervention

Two studies performed manual acupuncture (MA) [51, 52], and the rest of the studies utilized EA. The selection of acupoints was as follows: 29 reviews used ST36 monotherapy, 8 studies selected ST36 plus feishu (bladder meridian, BL13) [29, 31, 33, 34, 40, 45, 47, 48], 5 reviews selected ST36 plus shenshu (bladder meridian, BL23) [30, 37, 38, 43, 44], 4 studies chose ST36 plus fengfu (governor vessel, GV16) [32, 39, 41, 46]; 3 reviews selected ST36 plus baihui (governor vessel, GV20) [72, 74, 77], the remaining 6 studies [35, 36, 42, 49, 62, 63] selected ST36 plus other acupoints.

Acupuncture pretreatment was applied in 27 studies [29–49, 51, 63, 72, 74, 77, 78], another 24 studies administrated acupuncture after sepsis model induction, and the remaining 3 studies [21, 52, 73] applied the acupuncture both before and after model induction in different groups.

Time duration for acupuncture stimulation in the included studies varied from 12 min to 1.5 h. Among those 54 studies, 36 studies performed the acupuncture stimulation for 30 min, 3 articles [67, 70, 71] performed for 12 min, 6 articles [21, 29, 30, 32, 39, 46] performed for 15 min, 5 articles [37, 38, 59, 61] performed for 1h, 2 articles [53, 68] performed for 1.5 h, while 1 article performed for 20 min [69] and 45 min [49], respectively.

25 articles only performed acupuncture treatments for one time, 3 articles [67, 70, 71] for 6 times, 3 articles [57, 58, 62] for 3 times, and 21 articles [29–48, 78] for 5 times, while 2 articles [21, 73] performed different times of acupuncture treatment in different TG.

Bipolar waveform (BW) was used in 22 studies [29–49, 80] to performed stimulation in EA, continuous waveform (CW) in 1 study [62], and periodic waveform (PW) in 2 studies [57, 58]; all the three waveforms were used to treat and compared in different TG in 3 studies [72, 74, 77], and the remaining 24 studies did not mention the waveform in EA stimulation. The frequency applied in EA treatment in the studies varied from 2 Hz to 100 Hz. 2/100 Hz with BW were used in 23 studies [31, 33–35, 42, 44, 45, 53–56, 59–61, 64–66, 68, 73, 75, 76, 79, 81]. The maximum current used in the included studies was 40 mA, applied in 2 articles [49, 69]. The minimum current was adjusted to “induce a slight twitch of the limb” instead of a specific value, used in 3 articles [39, 46, 63]. However, 5 studies had no statement about the current [50, 62, 67, 70, 71] they used. 36 articles had no statement about the pulse width, which varied from 0.03 ms to 2 ms in the other 16 articles [21, 31, 33, 35, 36, 38, 42, 43, 45, 48, 49, 63, 67, 69, 70, 80]. Four articles [21, 64–66] described the value of the voltage, which is rarely a user-modifiable option on EA devices.

TG was compared with no treatment group as control group (CG) in 10 studies [49, 62, 66, 72–74, 76–78, 81], shame EA in 10 studies [21, 53–56, 59, 60, 65, 68, 79], and both in the remaining 34 studies.

#### 3.2.6. Supportive Therapies

26 studies reported details of supportive therapy in the articles. Among them, fluid resuscitation therapy [32, 39, 46, 54–61, 63, 64, 67, 69–74, 78–80] was used in 23 studies, keeping warm [32, 39, 46, 51, 52, 69] in 6 studies, and antibiotics in 1 study [21]. Fluid resuscitation was administrated with Lactated Ringer and normal saline (NS) with dosage varied from 30 mL/kg to 50 mL/kg by IV, IP, or hypodermic injection. The remaining 28 studies had no statement about supportive therapy in the articles.

#### 3.2.7. Quality of Studies and Publication Bias

Study quality checklist (SQC) scores varied from 2 to 10 out of a total of 17 points, with a median score of 6. Only 8 out of the 54 articles [34, 40, 44, 45, 64, 66, 74, 76] had statements about the control of temperature, humidity, and light. 50 articles reported using randomized allocation, none of which reported specific details of randomized allocation method. Only one study [52] reported details of allocation concealment. Three studies [21, 52, 63] reported using blinded outcome assessment, and 5 studies [32, 39, 40, 46, 74] used blinded assessment partly. No study reported using blinded model induction method, blinded intervention administration, or being free of selective reporting. Only 2 articles [48, 63] declared sample size calculation or explanation, one [48] of which was hard to evaluate the correctness of calculating method from its statements. 14 studies reported an animal welfare regulation and compliance statement. Only one study [49] did not describe the statistical method. Three articles [21, 41, 80] reported all the outcomes graphically, while 14 articles [32, 39, 46, 49, 51–53, 63, 68, 69, 73, 74, 76, 78] reported them partially in a graphical way and partially in a numerical way, and the remaining 37 articles reported all the data numerically. Six articles [21, 39, 63, 69, 73, 74] made a potential conflict of interests' statement. None of the 54 studies completed follow-up of animals' survival rates for more than 7 days or utilized intention-to-treat analysis when dealing with lost-to-continue animal data. Study quality and risk of bias checklist are shown in Table 2.

### 3.3. Effectiveness

It is not suitable to carry out meta-analysis for the high heterogeneity and low methodological quality of the studies.

#### 3.3.1. Effects of Acupuncture at ST36 on Survival Rate in Experimental Sepsis

Four studies [49, 69, 73, 74, 78] reported acupuncture at ST36 significantly increased the survival rates in sepsis animals. The survival rates were evaluated within 36 h [73], 72 h [69], and 7 days [74, 78] after model induction, respectively, while one article evaluated the length of survival time [49] within 10 hours.

#### 3.3.2. Effects of Acupuncture at ST36 on Cardiac Function in Experimental Sepsis

Four studies reported that, there were no significant differences in mean arterial blood pressure (MAP) and heart rate (HR) between TGs and the control groups (CGs), whether it was monitored by a small animal tail-cuff blood pressure analyzer [51, 52] or by an arterial catheter inserted into the artery [49, 63] in rats. However, in another research with rabbits [39], there was significant improvement in MAP and HR when comparing TG with CG. One study [65] reported acupuncture at ST36 significantly improved left ventricular end diastolic pressure (LVEDP), left ventricular end systolic pressure (LVSP), left ventricular pressure maximum rising rate (dp/dt max), cardiac output (CO), and HR, determined by the left cardiac catheterization from the pulmonary vein. Song et al. [68] and Zhang et al. [79] reported that acupuncture therapy could prevent myocardial damage during sepsis by detecting the plasma activity of creatine kinase-MB (CK-MB). EA at ST36 could also significantly lowered the ratios of the water content of heart tissue, indicating the potential of reducing inflammation and edema [68], The effects of upregulating the expression of heme oxygenase-1(HO-1) protein and mRNA in heart tissue might be the mechanism of the protective effect of inhibiting inflammation [35].

#### 3.3.3. Protective Effects of Acupuncture at ST36 on Lung Injury in Experimental Sepsis

Thirteen studies [29, 31–34, 37, 39–42, 44, 45, 48, 78] described significant lower histopathological grading when sepsis animals were treated with acupuncture, as well as lower wet-to-dry ratio (W/D) [32–34, 36–42, 44, 51], higher oxygenation indexes [34, 39, 40, 44], and less bronchoalveolar lavage fluid (BALF) albumin concentrations [39], indicating that acupuncture at ST36 might be effective in reducing sepsis-induced lung injury. The effect of suppressing mRNA expression of oxidative stress (MDA [31, 32, 34, 36, 37, 39–42, 44, 45, 48]) and inflammatory cytokines (iNOS and myeloperoxidase (MPO)) [51] and upregulating the mRNA and protein expression of their inhibitor (HO-1 [29, 33, 34, 37, 39–41, 48], SOD [32–34, 36, 37, 39–42, 44, 45, 48], NF-E2 related factor 2 (Nrf2) [31, 34, 36, 39, 42, 44–46, 48], and p38 mitogen-activated protein kinase (p38MAPK) [31, 34, 44, 45]) in lung tissue might be the underlying mechanism of the protective effect.

#### 3.3.4. Effects of Acupuncture at ST36 Ameliorates Renal Injury in Experimental Sepsis

It was reported that acupuncture at ST36 could protect kidneys from sepsis-induced injury by reducing renal MDA [30, 43, 46], renal inflammatory cytokines (inducible nitric oxide synthase (iNOS) [52, 63], nuclear factor *κ*B (NF-*κ*B) [63], MPO [52, 60], and tumor necrosis factor-*α* (TNF-*α*) [60]), plasma levels of blood urea nitrogen (BUN) [30, 43, 46, 52, 63], and creatinine (Cr) [30, 43, 46, 52, 60, 63], improving renal histopathology scores [30, 43, 46], upregulating renal SOD [30, 43, 46], and HO-1 protein [30, 43, 46] expression.

#### 3.3.5. Effects of Acupuncture at ST36 Ameliorates Brain Injury in Experimental Sepsis

The treatment of acupuncture at the ST36 could decrease brain injury and improve cognitive dysfunction [72, 74, 77], by preventing microglial activation (downregulating toll-like receptor-4 (TLR-4) and NF-*κ*B expression) and attenuating inflammation (decreasing TNF-*α*, interleukin-6 (IL-6) levels in serum and hippocampus, and reducing neuron-specific enolase (NSE) level in serum [64]), oxidative stress (lower MDA, higher SOD, and catalase (CAT) in serum and hippocampus), and reducing apoptosis [62].

#### 3.3.6. Effects of Acupuncture at ST36 Mitigate Damaging Other Organ Systems in Experimental Sepsis

Acupuncture at ST36 exerted positive effects on immune barrier by increasing the percentage of T lymphocyte [73] and reducing lymphocyte apoptosis in thymus [58, 62] and spleen [57]. It was reported that acupuncture at ST36 significantly decreased alanine aminotransferase (ALT) [54, 60, 61, 68, 70] and aspartate aminotransferase (AST) [52, 70], attenuating sepsis-induced hepatic injury by reducing the tissue water content of liver [55, 61, 68] and suppressing hepatic inflammatory cytokines (iNOS [55, 70], MPO [55, 60], and TNF-*α* [55]). Acupuncture therapies were reported significantly decreasing intestinal microvascular permeability [54, 56, 60, 66, 68] and improving microcirculation [66, 73, 76, 80], protecting intestinal epithelial cells from inflammatory response in sepsis [54, 56, 60, 66, 76, 81].

#### 3.3.7. Potential Mechanisms of the Protective Effects of Acupuncture at ST36 in Experimental Sepsis

It was described that acupuncture therapies could reduce content and activity of MDA [72, 74, 77], increase SOD [72, 74, 77] activity, and upregulate CAT [74] in sepsis animals, indicating that acupuncture at ST36 might be able to reduce oxidative stress during sepsis. Also, acupuncture therapies were reported as being capable of decreasing secretion of IL-6 [21, 39, 69, 72, 74, 77], high mobility group protein box-1 (HMGB1) [66, 67, 69, 76, 81], TNF-*α* [21, 30, 31, 34, 35, 38–41, 46, 48, 50, 63, 66–71, 74, 76, 77], IL-10 [30, 31, 38, 46, 48, 50, 63, 68], and interferon-*γ* (IFN-*γ*) [71], demonstrating acupuncture at ST36's potential of reducing inflammation in sepsis. Also, acupuncture could play a positive role in neurotrophy, particularly in the hippocampus, by upregulating Ghrelin, which is essential for cognitive adaptation to changing environments and the process of learning [66, 76].

Chen et al. reported that acupuncture at ST36 significantly reduced expression of TLR4 and NF-*κ*B, suggesting a possible pathway involving TLR4 and NF-*κ*B in sepsis animals [74, 77] might be the underlying mechanism of acupuncture's beneficial role. Acupuncture at ST36 might play a protective effect on maintaining immune balance in sepsis animals by upregulating CA3+, CD4+, and CD8+ lymphocytes expression and restoring an approximately average level of CD4+/CD8+ ratio [73]. Rafael reported that electroacupuncture at ST36 could control systemic inflammation by inducing a vagal activation of dopamine decarboxylase, thus leading to the production of dopamine in the adrenal medulla, while dopamine inhibits cytokine production via dopaminergic type-1 receptors [21].

Outcome measures of the included studies are summarized in Table 3.

## 4. Discussion

To our knowledge, this is the first systematic review of acupuncture at ST36 for sepsis animal model of literatures in both English and Chinese. The present study indicated that acupuncture at ST36 could be useful in reducing sepsis-induced injuries in heat, lung, kidney, liver, gastrointestinal tract, and immune system. Moreover, its potential mechanisms for antisepsis might include decreasing oxidative stress and inflammation, improving microcirculatory disturbance, and maintaining immune balance during sepsis.

However, the heterogeneity among the included studies was significant, the effectiveness of acupuncture therapy might be affected. From the heterogeneity analysis, we can conclude the following implications for further research.

### 4.1. Firstly, the Heterogeneity of Acupuncture Therapy was One of the Most Critical Factors

Only 2 of the 54 studies conducted MA, and remaining 52 studies performed EA. According to TCM theory, acupuncture produces therapeutic effects by the retention of needles at acupoints through acquiring “*Deqi*” manually. *Deqi* is a specific needle sensation, referring to the response to stimulations such as the thrusting, lifting, or rotating of the needle after insertion. It has been asserted to be a criterion to determine the appropriateness of acupuncture stimulation [82, 83]. The physical stimulation of MA is the key factor of its effects. However, when it comes to EA, the influence factors will be complicated by electrical stimulation. It is reported that simple focal electrical stimulation on mouse colon, even without acupuncture needle insertion, could produce phased-locked calcium signals in myenteric neurons and produced colon contractions to improve gastrointestinal function [84]. Also, Rafael et al. demonstrated EA at the sciatic nerve controls systemic inflammation by inducing a vagal activation of dopamine decarboxylase, while EA with a wood toothpick did not inhibit cytokine levels [21]. The results mentioned above indicate that only electrical stimulation itself might have a therapeutic effect already. Furthermore, the EA parameters, including waveform, frequency, current, and the pulse width, which differed a lot in the included studies, can be an essential factor influencing EA efficacy and its mechanism [85–87]. At the same time, 48.1% (26/54) of the studies used ST36 plus other acupoints. ST36, based on the TCM theory, could harmonize the spleen and stomach, tonify and replenish the middle qi, unblock the meridian and free the collateral vessels, disperse wind and transform dampness, reinforce the healthy qi, and eliminate the pathogenic factors. Adding other acupoints, in TCM theory, could synergize to protect the multiple organs and systems, such as cardiac, brain, lungs, kidneys, and liver. However, whether it is a synergistic interaction or an antagonistic effect actually is still unclear. Therefore, further study of acupuncture should pay attention to exploring the appropriate acupuncture intervention and EA parameters to achieve the best efficiency according to different targets.

### 4.2. Secondly, the Heterogeneity of Sepsis Animal Models was Another Factor

The animal model for sepsis in the included studies mainly lies in the LPS injection model and CLP model. LPS and CLP models could have similar mortality but significant differences in the kinetics and magnitude of cytokine production. LPS injection model is notable for the advantages of technical simplicity and high reproducibility, particularly in the eliciting of inflammatory response [88]. However, LPS injection model does not precisely reproduce the characteristic features of human sepsis, with shorter cytokine responses in duration than in humans [89].

CLP model, which is the most widely used sepsis model, is recognized to have significant compatibility with human sepsis [90–92]. The main advantage of the CLP model is that the peritoneum is persistently inoculated with mixed microbial flora from the animal itself [93], whereas LPS transient injection had no such effects. However, the CLP model is difficult to control and standardize, when comparing with the LPS injection models. Additionally, affects from variations in surgical procedures and postoperative care should also be considered [94].

Besides, some neotype sepsis models to compensate the weaknesses of conventional sepsis model has been invented for better reproducing various sepsis physiological progressions [95, 96]. So further studies for experimental sepsis should take these factors into consideration and choose the most appropriate model.

### 4.3. Thirdly, the Quality of Study Design and Reporting Should be Optimized

The methodological quality of the included studies was generally low. Statement of animal welfare compliance and sample size calculation are essential factors for evaluating the quality of evidence [25], although they are not criteria for risk of bias in animal trials. Most of the included studies had no report of allocation to model induction, intervention administration, blinding of outcome assessors, and outcome assessment and, as a result, risk of observer bias may exist [97].

Furthermore, we have found out some deficits in reported outcome data of the included studies. All articles utilizing New Zealand white rabbits were written by the same research team [29–48]. There were little differences in items of outcome measure, with the same animal species, sepsis model, and sample size. Results were identical in two different studies in lung tissue wet-to-dry ratio (W/D) [36, 45], oxygenation index [34, 40], SOD contents [34, 40], MDA contents in the lung tissues [34, 40], and TNF-*α* [67, 71], indicating the above studies might be Salami publications from the same experiment. Nevertheless, we cannot evaluate the correctness of this speculations because we failed to access more information from the authors by sending e-mails.

Moreover, there are several limitations in this systematic review. First, we included literatures published in Chinese or English only in this systematic review, so selective bias might exist. Second, most of the studies were published articles (47 out of 54 studies). Few data were collected from unpublished source. As a result, the efficacy of acupuncture therapy might be overestimated due to publication bias. Third, the general methodological quality of included studies was poor, suggesting that the results should be interpreted with caution.

## 5. Conclusions

54 studies were identified from 7 databases in this systematic review to evaluate the efficacy of acupuncture at ST36 for sepsis. In experimental sepsis, acupuncture at ST36 has been reported to be effective in ameliorating systematic injuries induced by sepsis. Poor methodological quality and publication bias exist. Interpreting the positive results should be done carefully. As the use of acupuncture is endorsed by the National Institute of Health and the World Health Organization, acupuncture might be a supplementary strategy for systematic inflammation control. It is worthy of further clinical and experimental trials.

## Figures and Tables

**Table 1:** Model details of included trials.

Study (years)	Model (method)
Cao et al., [43]	5 mg/kg LPS, i.v.
Dong et al., [29]	0.5 mL (5 mg/kg) LPS, i.v.
Gao et al., [44]	5 mg/kg LPS, i.v.
Gao et al., [45]	5 mg/kg LPS, i.v.
Gong et al., [37]	5 mg/kg LPS, i.v.
Guo et al., [38]	5 mg/kg LPS, i.v.
Han et al., [48]	5 mg/kg LPS, i.v.
Shi et al., [36]	5 mg/kg LPS, i.v.
Song et al., [42]	5 mg/kg LPS, i.v.
Wang et al., [30]	5 mg/kg LPS, i.v.
Yu et al., [32]	0.5 mL (5 mg/kg) LPS, i.v.
Yu et al., [39]	0.5 mL (5 mg/kg) LPS, i.v.
Yu et al., [46]	0.5 mL (5 mg/kg) LPS, i.v.
Zhang et al., [40]	0.5 mL (5 mg/kg) LPS, i.v.
Zhang et al., [34]	0.5 mL (5 mg/kg) LPS, i.v.
Zhang et al., [33]	5 mg/kg LPS, i.v.
Zhang et al., [31]	5 mg/kg LPS, i.v.
Zhang et al., [35]	2mL (5 mg/kg) LPS, i.v.
Zhang et al., [41]	2mL (5 mg/kg) LPS, i.v.
Zhang et al., [47]	5 mg/kg LPS, i.v.
Cao et al., [75]	CLP, no details
Chen et al., [74]	CLP, ligated 50% of the cecum with no. 0 silk, perforated once with 18-gauge needles (two pores)
Chen et al., [77]	CLP, ligated 50% of the cecum with no. 0 silk, perforated once with 18-gauge needles (two pores)
Gu et al., [63]	5 mg/kg LPS i.v.
Guo et al., [62]	CLP, ligated the cecum with no. 30 silk, perforated once with 22-gauge needles (two pores)
Hu et al., [53]	5 mg/kg LPS i.v.
Hu et al., [54]	CLP, ligature the root of the cecum, perforated 3 times with a no. 16 needle
Hu et al., [56]	CLP, no details
Hu et al., [55]	CLP, ligature the root of the cecum, perforated 3 times with a no. 16 needle
Hu et al., [60]	CLP, ligature the root of the cecum, perforated 3 times with a no. 16 needle
Huang et al., [51]	20 mg/kg LPS, intraperitoneally
Huang et al., [52]	20 mg/kg LPS, intraperitoneally
Yue et al., [67]	20% of the TBSA III degree burns on the back of the rat by boiling water (99°C–100°C, 12 s), following by 5 mg/kg MDP i.v.
Lei et al., [57]	CLP, ligated the cecum with no. 30 silk, perforated once with 22-gauge needles (two pores)
Lei et al., [58]	CLP, ligated the cecum with no. 30 silk, perforated once with 22-gauge needles (two pores)
Lei et al., [72]	CLP, ligated 50% of the cecum with no. 0 silk, perforated with 18-gauge needles (two pores)
Shi et al., [61]	CLP, ligature the root of the cecum, perforated 3 times with a no. 16 needle
Song et al., [68]	5 mg/kg LPS, i.v.
Song et al., [70]	20% of the TBSA III degree burns on the back of the rat by boiling water (99°C–100°C, 12 s), following by 5 mg/kg MDP i.v.
Villegas-Bastida et al., [69]	CLP, ligated just proximal to the ileocecal valve, perforated twice with 21-gauge needles at 5 mm distal to the point of ligation
Wang et al., [49]	30 mg/kg LPS, i.v.
Wang et al., [64]	CLP, ligated the root of the cecum, perforated 3 times with a no. 16 needle
Wang et al., [78]	CLP, ligated 1/3 of the cecum from the ileocecal valve with no. 0–4 silk, perforated with 18-gauge needles
Wu et al., [66]	CLP, ligated the root of the cecum, perforated 4 times with a no. 16 needle
Wu et al., [81]	CLP, ligated the root of the cecum, perforated 4 times with a no. 16 needle
Wu et al., [76]	CLP, ligated the root of the cecum, perforated 4 times with a size 16 needle
Xu et al., [65]	CLP, no details
Yang et al., [50]	CLP, ligated the root of the cecum, perforated 2 pores with a no. 12 needle
Zhang et al., [59]	CLP, ligated the root of the cecum, perforated 3 times with a size 16 needle
Zhang et al., [71]	20% of the TBSA III degree burns on the back of the rat by boiling water (99°C–100°C, 12 s), following 5 mg/kg MDP i.v.
Zhang et al., [79]	CLP, ligated the root of the cecum, perforated 3 times with no. 16 needle
Zhang et al., [80]	CLP, ligated the lower section of the cecum (25% from the blind side of its total length) using 4–0 silk threads, perforated once with a 19-gauge needle
Zhu et al., [73]	CLP, ligated the base of the cecum, below the ileocecal valve, with a 2.0-silk, perforated 2 times with no.18 needle
Torres-Rosas et al., [21]	(1) 6 mg/kg LPS, intraperitoneally (2) CLP, ligated the cecum at 5.0 mm from the cecal tip away from the ileocecal valve, punctured only once with a 22-gauge needle

*Note.* LPS: lipopolysaccharide; i.v.: intravenously; CLP: cecal ligation and puncture; TBSA: total body surface area; MDP : muramyl dipeptide.

**Table 2:** Study quality and risk of bias.

Study (years)	(1)	(2)	(3)	(4)	(5)	(6)	(7)	(8)	(9)	(10)	(11)	(12)	(13)	(14)	(15)	(16)	(17)	Total				Scores
																		+	±	?	–	
Cao et al., [43]	+	±	+	±^◆^	–	–	–	–	–	–	–	–	+	+	+	–	–	5	2	0	10	6
Dong et al., [29]	+	±	+	±^◆^	–	–	–	–	–	–	–	–	+	+	+	–	–	5	2	0	10	6
Gao et al., [44]	+	+	+	±^◆^	–	–	–	–	–	–	–	–	+	+	+	–	–	6	1	0	10	6.5
Gao et al., [45]	–^△^	+	+	±^◆^	–	–	–	–	–	–	–	–	+	+	+	–	–	5	1	0	11	5.5
Gong et al., [37]	+	±	+	±^◆^	–	–	–	–	–	–	–	–	+	+	+	–	–	5	2	0	10	6
Guo et al., [38]	+	–	+	±^◆^	–	–	–	–	–	–	–	–	+	+	+	–	–	5	1	0	11	5.5
Han et al., [48]	+	±	+	±^◆^	–	–	–	–	+	–	–	–	+	+	+	–	–	6	2	0	9	7
Shi et al., [36]	+	±	+	±^◆^	–	–	–	–	–	–	–	–	+	+	+	–	–	5	2	0	10	6
Song et al., [42]	+	±	+	±^◆^	–	–	–	–	–	–	–	–	+	+	+	–	–	5	2	0	10	6
Wang et al., [30]	–^△^	±	+	±^◆^	–	–	–	–	–	–	–	–	+	+	±	–	–	3	3	0	11	4.5
Yu et al., [32]	+	±	+	–	–	–	–	±	–	+	–	–	+	+	–	–	–	5	2	0	10	6
Yu et al., [39]	+	±	+	–	–	–	–	±	–	+	–	+	+	±	+	–	–	6	3	0	8	7.5
Yu et al., [46]	+	±	+	–	–	–	–	±	–	+	–	–	+	±	+	–	–	5	3	0	9	6.5
Zhang et al., [40]	–^△^	+	+	±^◆^	–	–	–	±	–	–	–	–	+	+	+	–	–	5	2	0	10	6
Zhang et al., [34]	–^△^	+	+	±^◆^	–	–	–	–	–	–	–	–	+	+	+	–	–	5	1	0	11	5.5
Zhang et al., [33]	+	±	+	±^◆^	–	–	–	–	–	–	–	–	+	+	+	–	–	5	2	0	10	6
Zhang et al., [31]	+	±	+	±^◆^	–	–	–	–	–	–	–	–	+	+	+	–	–	5	2	0	10	6
Zhang et al., [35]	+	–	+	±^◆^	–	–	–	–	–	–	–	–	+	+	+	–	–	5	1	0	11	5.5
Zhang et al., [41]	+	–	+	–	–	–	–	–	–	+	–	–	+	–	+	–	–	5	0	0	12	5
Zhang et al., [47]	+	–	+	±^◆^	–	–	–	–	–	–	–	–	+	+	+	–	–	5	1	0	11	5.5
Cao et al., [75]	+	–	+	–	–	–	–	–	–	–	–	–	+	+	+	–	–	5	0	0	12	5
Chen et al., [74]	+	+	+	–	–	–	–	±	–	+	–	+	+	±	+	–	–	7	2	0	8	8
Chen et al., [77]	+	–	+	–	–	–	–	–	–	–	–	–	+	+	+	–	–	5	0	0	12	5
Gu et al., [63]	+	±	+	±^◆^	–	–	–	+	+	+	–	+	+	±	+	–	–	8	3	0	6	9.5
Guo et al., [62]	+	–	+	±^◇^	–	–	–	–	–	–	–	–	+	+	+	–	–	5	1	0	11	5.5
Hu et al., [53]	+	–	+	–	–	–	–	–	–	–	–	–	+	±	–	–	–	3	1	0	13	3.5
Hu et al., [54]	+	–	+	±^◆^	–	–	–	–	–	–	–	–	+	+	+	–	–	5	1	0	11	5.5
Hu et al., [56]	+	–	+	±^◆^	–	–	–	–	–	–	–	–	+	+	+	–	–	5	1	0	11	5.5
Hu et al., [55]	+	–	+	±^◆^	–	–	–	–	–	–	–	–	+	+	+	–	–	5	1	0	11	5.5
Hu et al., [60]	+	–	+	±^◆^	–	–	–	–	–	–	–	–	+	+	+	–	–	5	1	0	11	5.5
Huang et al., [51]	+	±	+	–	–	–	–	–	–	+	–	–	+	±	–	–	–	4	2	0	11	5
Huang et al., [52]	+	–	+	±^◆^	+	–	–	+	–	+	–	–	+	±	–	–	–	6	2	0	9	7
Yue et al., [67]	+	–	+	±^◆^	–	–	–	–	–	–	–	–	+	+	+	–	–	5	1	0	11	5.5
Lei et al., [57]	+	–	+	±^◇^	–	–	–	–	–	–	–	–	+	+	+	–	–	5	1	0	11	5.5
Lei et al., [58]	+	–	+	±^◇^	–	–	–	–	–	–	–	–	+	+	–	–	–	4	1	0	12	4.5
Lei et al., [72]	–^△^	–	+	±^◆^	–	–	–	–	–	–	–	–	+	+	+	–	–	4	1	0	12	4.5
Shi et al., [61]	+	±	+	±^◆^	–	–	–	–	–	–	–	–	+	+	+	–	–	5	2	0	10	6
Song et al., [68]	+	±	+	–	–	–	–	–	–	–	–	–	+	±	–	–	–	3	2	0	12	4
Song et al., [70]	+	–	+	±^◆^	–	–	–	–	–	–	–	–	+	+	+	–	–	5	1	0	11	5.5
Villegas-Bastida et al., [69]	+	±	–	–	–	–	–	–	–	+	–	+	+	±	+	–	–	5	2	0	10	6
Wang et al., [49]	–^△^	±	–	–	–	–	–	–	–	–	–	–	–	±	+	–	–	1	2	0	14	2
Wang et al., [64]	+	+	+	±^◆^	–	–	–	–	–	?	–	–	+	+	+	–	–	6	1	1	9	7
Wang et al., [78]	+	±	+	±^◆^	–	–	–	–	–	–	–	–	+	±	–	–	–	3	3	0	11	4.5
Wu et al., [66]	–^△^	+	+	–	–	–	–	–	–	–	–	–	+	+	+	–	–	5	0	0	12	5
Wu et al., [81]	+	–	+	±^◆^	–	–	–	–	–	–	–	–	+	+	–	–	–	4	1	0	12	4.5
Wu et al., [76]	+	+	+	±^◆^	–	–	–	–	–	+	–	–	+	±	+	–	–	6	2	0	9	7
Xu et al., [65]	+	–	+	–	–	–	–	–	–	–	–	–	+	+	+	–	–	5	0	0	12	5
Yang et al., [50]	+	–	+	±^◆^	–	–	–	–	–	–	–	–	+	+	+	–	–	5	1	0	11	5.5
Zhang et al., [59]	+	±	+	±^◆^	–	–	–	–	–	–	–	–	+	+	–	–	–	4	2	0	11	5
Zhang et al., [71]	+	–	+	–	–	–	–	–	–	–	–	–	+	+	+	–	–	5	0	0	12	5
Zhang et al., [79]	+	±	–	–	–	–	–	–	–	+	–	–	+	+	+	–	–	5	1	0	11	5.5
Zhang et al., [80]	+	±	+	±^◆^	–	–	–	–	–	+	–	–	+	–	+	–	–	5	2	0	10	6
Zhu et al., [73]	+	±	–	–	–	–	–	–	–	+	–	+	+	±	+	–	–	5	2	0	10	6
Torres-Rosas et al., [21]	+	±	+	–	–	–	–	+	–	+	–	+	+	–	+	–	–	7	1	0	9	7.5

*Note.* (1) Peer-reviewed publication; (2) control of temperature, humidity, and light; (3) randomized allocation; (4) reporting details of randomized allocation method; (5) allocation concealment; (6) blinded model induction; (7) blinded intervention administration; (8) blinded outcome assessment; (9) sample size calculation or explanation; (10) animal welfare regulations compliance statement; (11) being free of selective reporting; (12) potential conflict of interests statement; (13) reporting statistical method; (14) reporting numerical data in Results; (15) reporting actual numbers of animal samples of different groups in Results; (16) completeness of follow-up; and (17) intention-to-treat analysis. +: yes, scores 1 point; –: no, scores 0 points; ±: partially yes, scores 0.5 points; ?: unclear, scores 0 points; ^△^unpublished Ph.D.'s or Master's thesis; ^◇^randomized allocation according to blocked randomization; details are unavailable; ^◆^randomized allocation according to random number table; details are unavailable.

**Table 3:** Characteristics of the included studies.

Study (years)	Outcome index	Intergroup differences^▽^
Cao et al., [43]	(1) Renal injury scores(2) BUN(3) Cr(4) MDA (renal)(5) SOD (renal)(6) PKC*α* (renal)(7) HO-1 (renal)(8) Nrf2 nucleoprotein relative expressions (renal)(9) Nrf2 total protein relative expressions (renal)	All *P* *<* 0.05^△◇^
Dong et al., [29]	(1) Lung injury scores(2) AI (alveolar epithelial cell)(3) HO-1(lung)(4) HO-1 mRNA (lung)	All *P* *<* 0.01^△^, NA^◇^
Gao et al., [44]	(1) Oxygenation Indexes(2) Lung injury scores(3) W/D (lung)(4) MDA (lung)(5) SOD (lung)(6) p38MAPK (lung)(7) p38MAPK phosphorylation (lung)(8) Nrf2 nucleoprotein relative expressions (lung)(9) Nrf2 total protein relative expressions (lung)(10) Nrf2 mRNA (lung)	All *P* *<* 0.05^△◇^
Gao et al., [45]	(1) Lung injury scores(2) W/D (lung)(3) SOD (lung)(4) MDA (lung)(5) p38MAPK (lung)(6) Nrf2 mRNA (lung)(7) Nrf2 total protein relative expressions (lung)	All *P* *<* 0.05^△◇^
Gong et al., [37]	(1) Lung injury scores(2) W/D (lung)(3) MDA (lung)(4) SOD (lung)(5) HO-1 mRNA (lung)(6) Nrf2 total protein relative expressions (lung)	(1) *P* *<* 0.05^△◇^(2) *P* *<* 0.05^△◇^(3) *P* *<* 0.05^△◇^(4) *P* *<* 0.05^△◇^(5) *P* *<* 0.05^△^, NA^◇^(6) *P* *<* 0.05^△^, NA^◇^
Guo et al., [38]	(1) Renal injury scores(2) W/D (renal)(3) *α*1-M (urine)(4) Nrf2 nucleoprotein relative expressions (renal)(5) Nrf2 total protein relative expressions (renal)(6) HO-1(renal)(7) Nrf2 mRNA (renal)(8) HO-1 mRNA (renal)	All *P* *<* 0.05^△◇^
Han et al., [48]	(1) Lung injury scores(2) SOD (lung)(3) MDA (lung)(4) TNF-*α*(5) IL-10(6) P-Akt protein (lung)(7) HO-1 (lung)(8) Nrf2 nucleoprotein relative expressions (lung)(9) Nrf2 total protein relative expressions (lung)	All *P* *<* 0.05^△◇^
Shi et al., [36]	(1) Lung injury scores(2) W/D (lung)(3) SOD (lung)(4) MDA (lung)(5) Nrf2 mRNA (lung)(6) Nrf2 nucleoprotein relative expressions (lung)(7) Nrf2 total protein relative expressions (lung)	All *P* *<* 0.05^△^, NA^◇^
Song et al., [42]	(1) Lung injury scores(2) W/D (lung)(3) MDA (lung)(4) SOD (lung)(5) Nrf2 nucleoprotein relative expressions (lung)(6) Nrf2 total protein relative expressions (lung)(7) PKC*α* (lung)(8) Nrf2 mRNA (lung)	All *P* *<* 0.05^△◇^
Wang et al., [30]	(1) Renal injury scores(2) AI (renal)(3) BUN(4) Cr(5) TNF-*α*(6) IL-10(7) *α*1-MG (urine)(8) MDA (renal)(9) SOD (renal)(10) HO-1 (renal)	All *P* *<* 0.05^△◇^
Yu et al., [32]	(1) SOD (lung)(2) MDA (lung)(3) EB contents (lung)(4) W/D (lung)(5) CO(6) Lung injure score(7) HO-1 mRNA(8) HO-1	All *P* *<* 0.05^△^, NA^◇^
Yu et al., [39]	(1) The death rate (the animals were supplemented)(2) MAP after EA for 30 min(3) MAP after LPS for 30 min(4) MAP after LPS for 60 min(5) MAP after LPS for 90 min(6) MAP after LPS for 120 min(7) Oxygenation indexes(8) W/D (lung)(9) MDA (lung)(10) SOD (lung)(11) CAT(12) GPx(13) TNF-*α*(14) IL-6(15) Leukocyte counts in the BALF(16) Albumin concentrations in the BALF(17) Lung injury scores(18) HO-1 mRNA (lung)(19) Nrf2 mRNA (lung)(20) HO-1 protein relative expressions (lung)(21) Nrf2 nucleoprotein relative expressions (lung)(22) Nrf2 total protein relative expressions (lung)	(1) NA^△^, NA^◇^(2) *P* *<* 0.05^△^, NA^◇^(3) *P* *<* 0.05^△^, NA^◇^(4) *P* *<* 0.05^△^, NA^◇^(5) *P* *<* 0.05^△^, NA^◇^(6) *P* *<* 0.05^△^, NA^◇^(7) *P* *<* 0.05^△^, NA^◇^(8) *P* *<* 0.05^△^, NA^◇^(9) *P* *<* 0.05^△^, NA^◇^(10) *P* *<* 0.05^△^, NA^◇^(11) *P* *<* 0.05^△^, NA^◇^(12) *P* *<* 0.05^△^, NA^◇^(13) *P* *<* 0.05^△^, NA^◇^(14) *P* *<* 0.05^△^, NA^◇^(15) *P* *<* 0.05, NA^◇^(16) *P* *<* 0.05^△^, NA^◇^(17) *P* *<* 0.05^△^, NA^◇^(18) *P* *<* 0.05^△^, NA^◇^(19) *P* *<* 0.05^△^, NA^◇^(20) *P* *<* 0.05^△^, NA^◇^(21) *P* *<* 0.05^△^, NA^◇^(22) *P* *<* 0.05^△^, NA^◇^
Yu et al., [46]	(1) SOD (renal)(2) MDA (renal)(3) IL-10(4) TNF-*α*(5) BUN(6) Cr(7) N-Acetylglucosaminidase(8) Renal injury scores(9) p-Akt protein (renal)(10) HO-1 (renal)(11) Nrf2 nucleoprotein relative expression (renal)(12) Nrf2 total protein relative expressions (renal)	All *P* *<* 0.05^△◇^
Zhang et al., [40]	(1) Lung injury scores(2) W/D (lung)(3) Oxygenation indexes(4) MDA (lung)(5) SOD (lung)(6) HO-1 (lung)(7) NF-κBp65 (lung)(8) HO-1 mRNA (lung)(9) TNF-*α*(10) HO-1 (lung)(11) ERK (lung)(12) p-ERK (lung)(13) HO-1 mRNA (lung)	(1) *P* *<* 0.05^△◇^(2) *P* *<* 0.05^△◇^(3) NA^△◇^(4) *P* *<* 0.05^△◇^(5) *P* *<* 0.05^△◇^(6) *P* *<* 0.05^△◇^(7) *P* *<* 0.05^△◇^(8) *P* *<* 0.05^△◇^(9) *P* *<* 0.05^△◇^(10) *P* *<* 0.05^△◇^(11) *P* *<* 0.05^△◇^(12) *P* *<* 0.05^△◇^(13) *P* *<* 0.05^△◇^
Zhang, [34]	(1) Lung injury scores(2) W/D (lung)(3) Oxygenation indexes(4) MDA (lung)(5) SOD (lung)(6) TNF-*α*(7) HO-1 (lung)(8) p38MAPK phosphorylation (lung)(9) HO-1 mRNA (lung)(10) PKC*α* mRNA (lung)	(1) *P* *<* 0.05^△◇^(2) *P* *<* 0.05^△◇^(3) NA^△◇^(4) *P* *<* 0.05^△◇^(5) *P* *<* 0.05^△◇^(6) *P* *<* 0.05^△◇^(7) *P* *<* 0.05^△◇^(8) *P* *<* 0.05^△◇^(9) *P* *<* 0.05^△◇^;
Zhang et al., [33]	(1) Lung injury scores(2) W/D (lung)(3) MDA (lung)(4) SOD (lung)(5) HO-1 mRNA (lung)(6) HO-1 (lung)(7) NF-κBp65total protein relative expressions (lung)(8) NF-κBp65 nucleoprotein relative expressions (lung)	All *P* *<* 0.05^△^, NA^◇^
Zhang et al., [31]	(1) Lung injury scores(2) TNF-*α*(3) IL-10(4) P38mapk (lung)	All *P* *<* 0.05^△^, NA^◇^
Zhang et al., [35]	(1) Cardiac injury scores(2) CK(3) LDH(4) TNF-*α*(5) HO-1 mRNA (cardiac)(6) HO-1 (cardiac)	All *P* *<* 0.05^△◇^
Zhang et al., [41]	(1) Lung injury score(2) W/D (lung)(3) SOD (lung)(4) MDA (lung)(5) TNF-*α*(6) HO-1 (lung)(7) HO-1 mRNA (lung)(8) ERK1/2 protein (lung)(9) p-ERK1/2 protein (lung)	All *P* *<* 0.05^△◇^
Zhang et al., [47]	(1) ATP (lung)(2) ROS (lung)(3) Mfn1 mRNA (lung)(4) Mfn2 mRNA (lung)(5) OPA1 mRNA (lung)(6) DRP1 mRNA (lung)(7) Mfn1 (lung)(8) Mfn2 (lung)(9) OPA1 (lung)(10) DRP1 (lung)	All *P* *<* 0.05^△◇^
Cao et al., [75]	(1) HR(2) LVEDP(3) LVSP(4) dp/dtmax(5) CO(6) TNF-*α* (cardiac)(7) IL-6 (cardiac)(8) MPO (cardiac)(9) S0D (cardiac)(10) MDA (cardiac)	(1) *P* *<* 0.01^△◇^(2) *P* *<* 0.01^△◇^(3) *P* *<* 0.01^△◇^(4) *P* *<* 0.01^△◇^(5) *P* *<* 0.01^△◇^(6) *P* *<* 0.05^△◇^(7) *P* *<* 0.05^△◇^(8) *P* *<* 0.01^△^, NA^◇^(9) *P* *<* 0.01^△^, NA^◇^(10) *P* *<* 0.01^△^, NA^◇^
Chen et al., [74]	(1) 7-day survival rate(2) The escape latency (MWM)(3) Retention Time (MWM)(4) Times across platform (MWM)(5) Swimming speed (MWM)(6) W/D (brain)(7) EB content (brain)(8) The total normal cell count of hippocampus(9) MDA(10) SOD(11) CAT(12) MDA (hippocampus)(13) SOD (hippocampus)(14) CAT (hippocampus)(15) IL-6(16) TNF-*α*(17) IL-6 (hippocampus)(18) TNF-*α* (hippocampus)(19) TLR-4 (hippocampus)(20) NF-κB (hippocampus)(21) Iba1 (hippocampus)	(1) *P* *<* 0.05^□^, *P* *<* 0.01^○☆^(2) *P* *<* 0.05^□^, *P* *<* 0.01^○☆^(3) *P* *<* 0.05^□^, *P* *<* 0.01^○☆^(4) *P* *<* 0.05^□^, *P* *<* 0.01^○☆^(5) *P* *>* 0.05^□○☆^(6) *P* *<* 0.05^□^, *P* *<* 0.01^○☆^(7) *P* *<* 0.05^□^, *P* *<* 0.01^○☆^(8) *P* *<* 0.05^□^, *P* *<* 0.01^○☆^(9) *P* *<* 0.05^□^, *P* *<* 0.01^○☆^(10) *P* *<* 0.05^□^, *P* *<* 0.01^○☆^(11) *P* *<* 0.05^□^, *P* *<* 0.01^○☆^(12) *P* *<* 0.05^□^, *P* *<* 0.01^○☆^(13) *P* *<* 0.05^□^, *P* *<* 0.01^○☆^(14) *P* *<* 0.05^□^, *P* *<* 0.01^○☆^(15) *P* *<* 0.05^□^, *P* *<* 0.01^○☆^(16) *P* *<* 0.05^□^, *P* *<* 0.01^○☆^(17) *P* *<* 0.05^□^, *P* *<* 0.01^○☆^(18) *P* *<* 0.05^□^, *P* *<* 0.01^○☆^
Chen et al., [77]	(1) The total normal cell count of hippocampus(2) AI (brain)(3) W/D (brain)(4) Expression of TLR-4 (brain)(5) TNF-*α*(6) IL-6(7) MDA(8) SOD(9) TNF-*α* (cortex)(10) IL-6 (cortex)(11) MDA (cortex)(12) SOD (cortex)	All *P* *<* 0.05^□○☆^
Gu et al., [63]	(1) TNF-*α*(2) IL-1β(3) IL-10(4) Nitrite(5) iNOS (renal)(6) NF-κB (renal) (7) BUN(8) Cr(9) Renal histopathological score(10) MAP(11) HR	(1) *P* *<* 0.001^△◇^(2) *P* *<* 0.05^△◇^(3) *P* *<* 0.001^△◇^(4) *P* *<* 0.05^△◇^(5) *P* *<* 0.05^△◇^(6) *P* *<* 0.05^△◇^(7) *P* *<* 0.05^△◇^(8) *P* *<* 0.05^△◇^(9) *P* *<* 0.05^△◇^(10) *P* *>* 0.05^△^, NA^◇^(11) *P* *>* 0.05^△^, NA^◇^
Guo et al., [62]	(1) Apoptosis rates of thymocytes(2) VIP (pituitary gland)(3) VIP	All *P* *<* 0.05^△^
Hu et al., [53]	CK-MB	*P* *<* 0.01^◇^
Hu et al., [54]	(1) TNF-*α* (jejunal)(2) NO (jejunal)(3) MP0 (jejunal)(4) DA0 (jejunal)(5) W/D (jejunal)	All *P* *<* 0.05^◇^
Hu et al., [56]	(1) JMBF(2) DAO (jejunal)(3) XOD (jejunal)(4) MDA (jejunal)(5) W/D (jejunal)	All *P* *<* 0.05^◇^
Hu et al., [55]	(1) ALT(2) TNF-*α* (liver)(3) NO (liver)(4) MPO (liver)(5) W/D (liver)	All *P* *<* 0.05^◇^
Hu et al., [60]	(1) ALT(2) Cr(3) DAO (liver, renal, and jejunal)(4) W/D (liver)(5) W/D (renal)(6) W/D (jejunal)(7) TNF-*α* (liver)(8) TNF-*α* (renal)(9) TNF-*α* (jejunal)(10) MPO (liver)(11) MPO (renal)(12) MPO (jejunal)	All *P* *<* 0.05^◇^
Huang et al., [51]	(1) Survival rate(2) HR(3) MAP(4) NO (lung)(5) NO(6) MPO (lung)(7) W/D (lung)(8) iNOS (lung) (RT-PCR)(9) iNOS (lung) (quantitative real-time PCR)(10) iNOS (lung) (immunoblotting)	(1) *P* *>* 0.05^☼^, NA*δ*(2) *P* *>* 0.05^☼^, NA*δ*(3) *P* *>* 0.05^☼^, NA*δ*(4) *P* *<* 0.05^☼^*δ*(5) *P>*0.05^☼^, NA*δ*(6) *P* *<* 0.05^☼^*δ*(7) *P>*0.05^☼^, NA*δ*(8) *P* *<* 0.05^☼^, *P>*0.05*δ*(9) *P* *<* 0.05^☼^*δ*(10) *P* *<* 0.05^☼^*δ*
Huang et al., [52]	(1) BUN(2) Cr(3) AST(4) ALT(5) Total bilirubin(6) HR(7) MAP(8) PMN (renal)(9) MPO (renal)(10) PMN (liver)(11) MPO (liver)(12) NO (renal)(13) NO (liver)14) iNOS mRNA (renal) (15) iNOS mRNA (liver)(16) iNOS protein (renal)(17) iNOS protein (liver)	(1) *P<*0.05^☼^, *P>*0.05φ(2) *P<*0.05^☼^φ(3) *P>*0.05^☼^φ(4) *P* *>* 0.05^☼^φ(5) *P* *>* 0.05^☼^φ(6) *P* *>* 0.05^☼^φ(7) *P* *>* 0.05^☼^φ(8) NA^☼^, *P* *<* 0.05φ(9) NA^☼^, *P* *<* 0.05φ(10) NA^☼^, *P* *>* 0.05φ(11) NA^☼^, *P* *>* 0.05φ(12) NA^☼^, *P* *<* 0.05φ(13) NA^☼^, *P* *>* 0.05φ(14) NA^☼^, *P* *<* 0.05φ(15) NA^☼^, *P* *>* 0.05φ(16) NA^☼^, *P* *<* 0.05φ(17) NA^☼^, *P* *>* 0.05φ
Yue et al., [67]	(1) NLR2 mRNA (lung)(2) RIP2 (lung)(3) TNF-*α*(4) HMGB1	All *P* *<* 0.05^△◇^
Lei et al., ^△^ [57]	(1) AI (splenic lymphocyte)(2) Bcl-2 protein (splenic lymphocyte)	All *P* *<* 0.05^△◇^
Lei et al., [58]	(1) AI (thymocyte)(2) VIP (pituitary gland)(3) VIP	All *P* *<* 0.05^△◇^
Lei et al., [72]	(1) The escape latency (MWM)(2) Times across platform (MWM)(3) Retention time (MWM)(4) TNF-*α*(5) IL-6(6) TNF-*α* (hippocampus)(7) IL-6 (hippocampus)(8) MDA (hippocampus)(9) SOD (hippocampus)(10) MDA(11) SOD(12) TLR-4 (cortex)(13) NF-Kb (cortex)(14) TLR-4 (hippocampus)(15) NF-Kb (hippocampus)(16) AI (hippocampus)(17) AI (cortex)	All *P* *<* 0.05^□○☆^
Shi et al., [61]	(1) HBF(2) ALT(3) MDA (liver)(4) XOD (liver)(5) W/D (liver)	All *P* *<* 0.05^△◇^
Song et al., [68]	(1) TNF-*α*(2) IL-10(3) ALT(4) CK-MB(5) Cr(6) DAO(7) W/D (cardiac)(8) W/D (liver)(9) W/D (kidney)(10) W/D (intestine)	All *P* *<* 0.05^◇^
Song et al., [70]	(1) ALT(2) AST(3) HMGB1(4) TNF-*α*(5) NLR2 mRNA (liver)(6) RIP2 mRNA (liver)	All *P* *<* 0.05^△^, NA^◇^
Villegas-Bastida et al., [69]	(1) TNF-*α* (2h)(2) TNF-*α* (6h)(3) TNF-*α* (18h)(4) IL-6 (2h)(5) IL-6 (6h)(6) IL-6 (18h)(7) Nitrite (2h)(8) Nitrite (6h)(9) Nitrite (18h)(10) HMGB1(2h)(11) HMGB1(6h)(12) HMGB1(18h)(13) 72h survival rate	(1) *P* *<* 0.05^△^, *P* *>* 0.05^◇^(2) *P* *<* 0.01^△^, *P* *>* 0.05^◇^(3) *P* *<* 0.05^△^, *P* *>* 0.05^◇^(4) *P* *<* 0.01^△^, *P* *>* 0.05^◇^(5) *P* *<* 0.05^△^, *P* *>* 0.05^◇^(6) *P* *<* 0.05^△^, *P* *>* 0.05^◇^(7) *P* *>* 0.05^△◇^(8) *P* *<* 0.05^△^, *P* *>* 0.05^◇^(9) *P* *<* 0.05^△^, *P* *>* 0.05^◇^(10) *P* *>* 0.05^△◇^(11) *P* *<* 0.01^△^, *P* *>* 0.05^◇^(12) *P* *<* 0.05^△^, *P* *>* 0.05^◇^(13) *P* *>* 0.05^△^, NA^◇^
Wang et al., [49]	(1) Survival time(2) bp (0 min, 30 min, 60 min, 90 min, 120 min, 150 min, 180 min, 210 min, 240 min, 270 min, 300 min)(3) Temperature (0 min, 60 min, 120 min, 180 min, 240 min, 300 min)(4) HR (0 min, 30 min, 60 min, 90 min, 120 min, 150 min, 180 min, 210 min, 240 min, 270 min, 300 min)	(1) *P* *<* 0.05^△^(2) NA^△^(3) NA^△^(4) NA^△^
Wang et al., [64]	(1) TNF-*α* (brain)(2) IL-6 (brain)(3) NSE	All *P* *<* 0.05^△◇^
Wang et al., [78]	(1) 7d survival rate(2) Lung injury scores(3) Liver injury scores(4) TNF-*α*(5) IL-1β(6) IL-6(7) HMGB1 (lung)	All *P* *<* 0.05^△^
Wu et al., [66]	(1) Chiu's scores (intestine)(2) W/D (intestine)(3) TNF-*α*(4) HMGB1(5) MPO (intestine)(6) DAO (intestine)(7) HMGB1 (intestine)(8) Ghrelin (intestine)(9) Ghrelin receptor (intestine)(10) Ghrelin(11) Ghrelin (immunohistochemistry)(12) Ghrelin receptor (immunohistochemistry)	All *P* *<* 0.05^△^
Wu et al., [81]	(1) HMGB1(2) Ghrelin(3) HMGB1 (intestine)(4) Ghrelin (intestine)	All *P* *<* 0.05^△^
Wu et al., [76]	(1) Ghrelin(2) Ghrelin (intestine)(3) GSH-R (intestine)(4) TNF-*α*(5) HMGB1(6) HMGB1 (intestine)(7) MPO (intestine)(8) DAO (intestine)(9) W/D (intestine)(10) Chiu's score (intestine)	All *P* *<* 0.05^△^
Xu et al., [65]	(1) HR(2) LVEDP(3) LVSP(4) dp/dtmax(5) CO(6) MMP-2 mRNA(cardiac)(7) MMP-9 mRNA (heart)(8) TIMP-1 mRNA (cardiac)(9) TIMP-2 mRNA (cardiac)	All *P* *<* 0.05^◇^
Yang et al., [50]	(1) TNF-*α*(2) IL-10	(1) *P* *<* 0.01^△◇^(2) *P* *>* 0.05^△◇^
Zhang et al., [59]	(1) CK-MB(2) TNF-*α* (cardiac)(3) NO (cardiac)(4) MPO (cardiac)(5) W/D (cardiac)	All *P* *<* 0.05^◇^
Zhang et al., [71]	(1) pH (arterial blood)(2) PaO_2_ (arterial blood)(3) lactate (arterial blood)(4) TNF-*α*(5) HMGB1(6) IFN-*γ*	All *P* *<* 0.05^△^, NA^◇^
Zhang et al., [79]	(1) CK-MB(2) TNF-*α* (cardiac)(3) NO (cardiac)(4) MPO (cardiac)(5) W/D (cardiac)	All *P* *<* 0.05^◇^
Zhang et al., [80]	(1) Chiu's score (intestine)(2) d-lactate(3) Occludin immunohistochemistry scores(4) Occludin protein expression	All *P* *<* 0.05^△^, *P* *>* 0.05^◇^
Zhu et al., [73]	(1) 36h survival rate(2) intestinal injury score(3) the circulating D-Lactose(4) sIgA content in intestinal mucosa cells(5) CD3+ T lymphocytes(6) *γ*/*δ*T lymphocytes(7) CD 4+ T lymphocytes(8) CD 8+ T lymphocytes(9) CD4+/CD8+ T lymphocytes	(1) *P* *<* 0.05^△^, *P* *>* 0.05*ψω*(2) *P* *<* 0.05^△^*ψω*(3) *P* *<* 0.01^△^ω, *P* *<* 0.05ψ(4) *P* *<* 0.05^△^ω, *P* *>* 0.05ψ(5) *P* *<* 0.05^△^ω, *P* *>* 0.05ψ(6) *P* *<* 0.05^△^*ψω*(7) *P* *<* 0.05^△^*ψω*(8) *P* *>* 0.05^△^*ψω*

*Note.* ^▽^A *p*-value of the comparison between the treatment group and control group at each test time phase unless otherwise stated; ^△^, EA versus S; ^◇^, EA versus S + SEA;^□^, CW versus S; ^○^, PW versus S; ^☆^, BW versus S; ^☼^, MA versus S; *δ*, MA versus S + SEA; φ, pMA versus S; ψ, low-frequency EA versus S; ω, high-frequency EA versus S; EA, electroacupuncture group; MA, manual acupuncture; S, sepsis model group; SEA, sepsis model plus shame EA group; pMA, manual acupuncture pretreatment; CW, continuous waveform; PW, periodic waveform; BW, bipolar waveform; BUN, blood urea nitrogen; Cr, creatinine; MDA, malondialdehyde; SOD, superoxide dismutase; PKC*α*, protein kinase Ca; HO-1, heme oxygenase-1; Nrf2, NF-E2 related factor 2; AI, apoptosis index; W/D, wet-to-dry ratio; p38MAPK, p38 mitogen-activated protein kinase; *α*1-M, *α*1-microglobulin; TNF-*α*, tumor necrosis factor-*α*; IL, interleukin; P-Akt, phosphorylated Akt; EB, Evans blue; CO, carbon monoxide; CAT, catalase; GPx, glutathione peroxidase; BALF, bronchoalveolar lavage fluid; ERK, extracellular signal-regulated kinases; CK, creatine kinase; LDH, lactate dehydrogenases; ATP, adenosine triphosphate; ROS, reactive oxygen species; Mfn, mitochondria fusion protein mitofusin; OPA, optic atrophic; Drp, dynamin-related protein; MAP, mean arterial blood pressure; HR, heart rate; LVEDP, left ventricular end diastolic pressure; LVSP, left ventricular end systolic pressure; dp/dt max, left ventricular pressure maximum rising rate; CO, cardiac output; iNOS, inducible nitric oxide synthase; VIP, vasoactive intestinal peptide; NO, nitric oxide; DAO, diamine oxidase; JMBF, the mucosal blood flow of jejunum; XOD, xanthine oxidase; PMN, polymorphonuclear neutrophil; NLR, Nod-like receptor; RIP, receptor interacting protein; HMGB1, high mobility group protein box-1; BCL-2, B-cell lymphoma 2; NF-κB, nuclear factor κB; HBF, hepatic blood flow; NSE, neuron-specific enolase; MMP, matrix metalloproteinase; TIMP, tissue inhibitor of metalloproteinase; pH, potential of hydrogen; IFN-*γ*, interferon-*γ*; MWM, Morris water maze; Iba, ionized calcium binding adaptor molecule 1.

## Appendix

### Search Strategy for MEDLINE (PubMed)

  #1 (ST36) OR (zusanli) OR (Tzusanli)
  #2 (Electro-acupuncture) OR (Electroacupuncture) OR (EA) OR (acupuncture) OR (acupuncture electric stimulation) OR (AES)
  #3 (Sepsis [Mesh]) OR (Septicemia) OR (bloodstream infection) OR (Blood stream infection) OR (Septic Shock) OR (Endotoxic Shock) OR (Toxic Shock) OR (Severe sepsis) OR (Endotoxin) OR (endotoxaemic)
  #4 ((randomized controlled trial [pt]) OR (controlled clinical trial [pt]) OR (randomized [tiab]) OR (placebo [tiab]) OR (randomly [tiab]) OR (trial [tiab]) OR (groups [tiab])) AND (animal [mh])
  #5 #1 AND #2 AND #3 AND #4

## References

[1] Kaukonen K. M., Bailey M., Pilcher D. (2015). Systemic inflammatory response syndrome criteria in defining severe sepsis. *The New England Journal of Medicine*.

[2] Iskander K. N., Osuchowski M. F., Stearns-Kurosawa D. J. (2013). Sepsis: multiple abnormalities, heterogeneous responses, and evolving understanding. *Physiological Reviews*.

[3] Cedervall J., Zhang Y., Olsson A. K. (2016). Tumor-induced NETosis as a risk factor for metastasis and organ failure. *Cancer Research*.

[4] Karasu E., Nilsson B., Kohl J. (2019). Targeting complement pathways in polytrauma- and sepsis-induced multiple-organ dysfunction. *Frontiers in Immunology*.

[5] Fleischmann C., Scherag A., Adhikari N. K. (2016). Assessment of global incidence and mortality of hospital-treated sepsis. Current estimates and limitations. *American Journal of Respiratory and Critical Care Medicine*.

[6] Angus D. C., van der Poll T. (2013). Severe sepsis and septic shock. *The New England Journal of Medicine*.

[7] Levy M. M., Dellinger R. P., Townsend S. R. (2010). The Surviving Sepsis Campaign: results of an international guideline-based performance improvement program targeting severe sepsis. *Intensive Care Medicine*.

[8] Cohen J., Opal S., Calandra T. (2012). Sepsis studies need new direction. *The Lancet Infectious Diseases*.

[9] Abraham E., Laterre P. F., Garbino J. (2001). Lenercept (p55 tumor necrosis factor receptor fusion protein) in severe sepsis and early septic shock: a randomized, double-blind, placebo-controlled, multicenter phase III trial with 1,342 patients. *Critical Care Medicine*.

[10] Vickers A., Zollman C. (1999). ABC of complementary medicine. Acupuncture. *BMJ*.

[11] Langevin H. M., Churchill D. L., Fox J. R. (2001). Biomechanical response to acupuncture needling in humans. *Journal of Applied Physiology*.

[12] Sekido R., Ishimaru K., Sakita M. (2003). Differences of electroacupuncture-induced analgesic effect in normal and inflammatory conditions in rats. *The American Journal of Chinese Medicine*.

[13] Zhang R. X., Lao L., Wang L. (2004). Involvement of opioid receptors in electroacupuncture-produced anti-hyperalgesia in rats with peripheral inflammation. *Brain Research*.

[14] Hu L., Klein J. D., Hassounah F. (2015). Low-frequency electrical stimulation attenuates muscle atrophy in CKD--a potential treatment strategy. *Journal of the American Society of Nephrology : JASN*.

[15] Du M. H., Luo H. M., Hu S. (2013). Electroacupuncture improves gut barrier dysfunction in prolonged hemorrhagic shock rats through vagus anti-inflammatory mechanism. *World Journal of Gastroenterology*.

[16] Lee M. J., Jang M., Choi J. (2016). Bee venom acupuncture alleviates experimental autoimmune encephalomyelitis by upregulating regulatory T cells and suppressing Th1 and Th17 responses. *Molecular Neurobiology*.

[17] Gao W., Huang Y. X., Chen H. (2000). Regulatory effects of electro-acupuncture at Zusanli on ir-SP content in rat pituitary gland and peripheral blood and their immunity. *World Journal of Gastroenterology*.

[18] Wang H., Pan Y., Xue B. (2011). The antioxidative effect of electro-acupuncture in a mouse model of Parkinson’s disease. *PloS One*.

[19] Ng S. S., Leung W. W., Mak T. W. (2013). Electroacupuncture reduces duration of postoperative ileus after laparoscopic surgery for colorectal cancer. *Gastroenterology*.

[20] Meng J. B., Jiao Y. N., Zhang G. (2018). Electroacupuncture improves intestinal dysfunction in septic patients: a randomised controlled trial. *BioMed Research International*.

[21] Torres-Rosas R., Yehia G., Pena G. (2014). Dopamine mediates vagal modulation of the immune system by electroacupuncture. *Nature Medicine*.

[22] Lalu M. M., Sullivan K. J., Mei S. H. (2016). Evaluating mesenchymal stem cell therapy for sepsis with preclinical meta-analyses prior to initiating a first-in-human trial. *eLife*.

[23] Murphy S. P., Murphy A. N. (2010). Pre-clinical systematic review. *Journal of Neurochemistry*.

[24] Wang W. W., Xie C. L., Lu L. (2014). A systematic review and meta-analysis of Baihui (GV20)-based scalp acupuncture in experimental ischemic stroke. *Scientific Reports*.

[25] Krauth D., Woodruff T. J., Bero L. (2013). Instruments for assessing risk of bias and other methodological criteria of published animal studies: a systematic review. *Environmental Health Perspectives*.

[26] Macleod M. R., O’Collins T., Howells D. W. (2004). Pooling of animal experimental data reveals influence of study design and publication bias. *Stroke*.

[27] Piper R. D., Cook D. J., Bone R. C. (1996). Introducing Critical Appraisal to studies of animal models investigating novel therapies in sepsis. *Critical Care Medicine*.

[28] Lai F., Zhang Y., Xie D. P. (2015). A systematic review of rhubarb (a traditional Chinese medicine) used for the treatment of experimental sepsis. *Evidence-based Complementary and Alternative Medicine*.

[29] Dong S., Luo X., Yu J. (2012). Effects of electro-acupuncture at Zusanli and Feishu on endotoxin shock-induced acute lung injury in rabbits Chinese. *Journal of Anesthesiology*.

[30] Wang B. (2013). Effects of Electro-acupuncture at Zusanli and Shenshu in Endotoxin-induced acute kidney injury in rabbits.

[31] Zhang G., Yu J., Gong L. (2013). Role of p38 mitogen-activated protein kinase pathway in electro-acupuncture-induced reduction of endotoxic shock-induced acute lung injury in rabbits Chinese. *Journal of Anesthesiology*.

[32] Yu J. B., Dong S. A., Luo X. Q. (2013). Role of HO-1 in protective effect of electro-acupuncture against endotoxin shock-induced acute lung injury in rabbits. *Experimental Biology and Medicine*.

[33] Zhang Y., Yu J., Gong L. (2013). Role of nuclear factor-kappaB in electro-acupuncture-induced up-regulation of heme oxygenase-1 expression in rabbits with endotoxic shock-induced acute lung injury Chinese. *Journal of Anesthesiology*.

[34] Zhang G. (2014). Effect of PKC*α* and p38MAPK signaling pathwany in electroacupunture-mediated up-regulation of heme oxygenase-1 in rabbits with endotoxic shock induced acute lung injury.

[35] Zhang G., Yu J. (2014). Effect of electro-acupuncture at Zusanli and Neiguan acupoints on endotoxic shock-induced myocardial injury in rabbits and the role of heme oxygenase-1 Chinese. *Journal of Anesthesiology*.

[36] Shi J., Yu J., Gong L. (2014). Mechanism of electroacupuncture-induced reduction of acute lung iniury induced by endotoxic shock in rabbits: the relationship with Nrf2/ARE pathway Chinese. *Journal of Anesthesiology*.

[37] Gong L., Yu J., Xu Y. (2014). Role of activator protein-1 in electro-acupuncture-induced up-regulation of heme oxygenase-1 expression in lung tissues in a rabbit model of endotoxic shock. *Chinese Journal of Anesthesiology*.

[38] Guo Y., Gong L., Yu J. (2014). Effects of electro-acupuncture on endotoxic shock-induced acute kidney injury in rabbits: relationship with Keap1-Nrf2/ARE signaling pathway. *Chinese Journal of Anesthesiology*.

[39] Yu J. B., Shi J., Gong L. R. (2014). Role of Nrf2/ARE pathway in protective effect of electroacupuncture against endotoxic shock-induced acute lung injury in rabbits. *PloS One*.

[40] Zhang Y. (2014). Effect of ERK1/2 and NF-*κ*B signaling pathway in electroacupuncture-mediated up-regulation of heme oxygenase-1 in lung of rabbits with endotoxic shock.

[41] Zhang Y., Yu J. B., Luo X. Q. (2014). Effect of ERK1/2 signaling pathway in electro-acupuncture mediated up-regulation of heme oxygenase-1 in lungs of rabbits with endotoxic shock. *Medical Science Monitor : International Medical Journal of Experimental and Clinical Research*.

[42] Song K., Yu J., Gong L. (2015). Effect of protein kinase C on Nrf2 expression in rabbits with endotoxin-induced lung injury induced by electroacupuncture. *Chinese Journal of Emergency Medicine*.

[43] Cao X., Shi J., Yu J. (2015). Role of PKC*α* in electroacupuncture-induced reduction of acute kidney injury induced by endotoxic shock in rabbits: the relationship with Nrf2/HO-1 pathway Chinese. *Journal of Anesthesiology*.

[44] Gao X. (2015). The Effect of p38MAPK in affecting Keap1-Nrf2/ARE pathway in protective Effect of electroacupuncture against endotoxin shock induced ALI in rabbits.

[45] Gao X., Gong L., Yu J. (2015). Role of p38MAPK signaling pathway in electroacupuncture-induced reduction of ALI in rabbits with endotoxic shock: the relationship with Nrf2 Chinese. *Journal of Anesthesiology*.

[46] Yu J. B., Shi J., Zhang Y. (2015). Electroacupuncture ameliorates acute renal injury in lipopolysaccharide-stimulated rabbits via induction of HO-1 through the PI3K/Akt/Nrf2 pathways. *PloS One*.

[47] Zhang Y., Yu J. (2018). Effects of electroacupuncture on mitochondrial fusion-fission during endotoxin-induced acute lung injury in rabbits Chinese. *Journal of Anesthesiology*.

[48] Han Y., Shi J., Wu L. (2018). Effect of PI3K/Akt/Nrf2 signaling pathway on electroacupuncture-induced reduction of acute lung injury induced by endotoxic shock in rabbits Chinese. *Journal of Surgery of Integrated Traditional and Western Medicine*.

[49] Wang H. (2004). Effect of electroacupuncture at zusanli point on endotoxin shock and chemical gastric ulcer in rats.

[50] Yang C., Yuan X., Li J. (2006). Effects of electroacupuncturing “zusanli” points on levels of TNF-*α* and IL-10 in septic shock rats. *Journal of New Medicine*.

[51] Huang C. L., Huang C. J., Tsai P. S. (2006). Acupuncture stimulation of ST-36 (Zusanli) significantly mitigates acute lung injury in lipopolysaccharide-stimulated rats. *Acta Anaesthesiologica Scandinavica*.

[52] Huang C. L., Tsai P. S., Wang T. Y. (2007). Acupuncture stimulation of ST36 (Zusanli) attenuates acute renal but not hepatic injury in lipopolysaccharide-stimulated rats. *Anesthesia and Analgesia*.

[53] Hu S., Song Q., Shi D. (2008). Protective effect of cholinergic pathway excitation on heart injury after endotoxin challenge in rats infection. *Inflammation and Repair*.

[54] Hu S., Zhang L., Bai H. (2009). Effects of electro-acupuncture at Zusanli point on the expression of proinflammatory cytokines, the activity of diamine oxidase and the rate of water content in the small intestine in rats with sepsis. *World Chinese Journal of Digestology*.

[55] Hu S., Zhang L., Bai H. (2009). The protective effect of electro-acupuncturing at zusanli point on proinflammatory factors induced-hepatic in rats with sepsis. *Journal of Medical Research*.

[56] Hu S., Zhang L., Bai H. (2009). The effects of eIectro-acupuncturing at Zusanli point on intestinal proinflammatory factors, dlamine oxidase and tissue water content in rats with sepsis Chinese Critical Care. *Medicine*.

[57] Lei S., Xu Y., Jiang R. (2009). Infuence of electro-acupuncture at tsusanli on the apoptosis of splenic lymphocytes and apoptosis-related gene expression in rats with sepsis. *Chinese Archives of Traditional Chinese Medicine*.

[58] Lei S., Xu Y., Jiang R. (2009). Effects of electro-acupuncture at “Zusanli” on the apoptosis of thymocytes in rats with sepsis. *China Journal of Traditional Chinese Medicine and Pharmacy*.

[59] Zhang L., Shi X., Bai H. (2010). Protetive effects of electroacupuncture at Zusanli point on myocardial injury induced by proinflammatory factors in rats. *Academic Journal of Chinese PLA Medical School*.

[60] Hu S., Zhang L., Bai H. (2010). Effect of electro-acupuncture at Zusanli point on tumor necrosis factor-*α* induced-multiple organ dysfunction in rats with sepsis. *Chinese Journal of Pathophysiology*.

[61] Shi X., Zhang L., Bai H. (2010). Effects of electroacupuncture on hepatic blood flow and lipid peroxidation in septic rats. *Chinese Acupuncture & Moxibustion*.

[62] Guo X., Zhu M., Xu Y. (2010). Effects of electroacupuncture at zusanli (ST36) and guanyuan (CV4) on the apoptosis of thymocytes in rats with sepsis. *Journal of Emergency in Traditional Chinese Medicine*.

[63] Gu G., Zhang Z., Wang G. (2011). Effects of electroacupuncture pretreatment on inflammatory response and acute kidney injury in endotoxaemic rats. *The Journal of International Medical Research*.

[64] Wang H., Du M., Shi X. (2013). Effects of acupuncture at “Zusanli” (ST36) on cerebral proinflammatory cytokine and plasma neuron specific enolase in septic rats. *Chinese Acupuncture & Moxibustion*.

[65] Xu T., Cao H., Xu M. (2013). Effect of electro-acupuncture at Zusanli point on the injury of the heart function in sepsis rats. *Chinese Journal of Experimental Surgery*.

[66] Wu J. (2014). Correlation between Expression of ghrelin and its receptor and Electroacupuncture intervention in rats with acute intestinal injury.

[67] Yue L., Song X., Zhang Z. (2014). Effect of electro-acupunetare at zusanli on acute lung injury in a rat model of sepsis after scald. *Chinese Journal of Anesthesiology*.

[68] Song Q., Hu S., Wang H. (2014). Electroacupuncturing at Zusanli point (ST36) attenuates pro-inflammatory cytokine release and organ dysfunction by activating cholinergic anti-inflammatory pathway in rat with endotoxin challenge. *African Journal of Traditional, Complementary, and Alternative Medicines : AJTCAM*.

[69] Villegas-Bastida A., Torres-Rosas R., Arriaga-Pizano L. A. (2014). Electrical stimulation at the ST36 acupoint protects against sepsis lethality and reduces serum TNF levels through vagus nerve- and catecholamine-dependent mechanisms. *Evidence-based Complementary and Alternative Medicine*.

[70] Song X., Yue L., Han Y. (2014). Effect of electro-acupunctare at Zusanli on liver injury in a rat model of sepsis after scald Chinese. *Journal of Anesthesiology*.

[71] Zhang C., Wang Y., Song X. (2015). Effect of electro-acupuncture at zusanli on thermal and muramyl dipeptide-induced sepsis in rats. *Chinese Journal of Experimental Surgery*.

[72] Lei Y. (2015). A study of protective Effect of pretreatment with different waveform electroacupuncture on cerebral injury and potential mechanism in sepsis rats.

[73] Zhu M. F., Xing X., Lei S. (2015). Electroacupuncture at bilateral zusanli points (ST36) protects intestinal mucosal immune barrier in sepsis. *Evidence-Based Complementary and Alternative Medicine*.

[74] Chen Y., Lei Y., Mo L. Q. (2016). Electroacupuncture pretreatment with different waveforms prevents brain injury in rats subjected to cecal ligation and puncture via inhibiting microglial activation, and attenuating inflammation, oxidative stress and apoptosis. *Brain Research Bulletin*.

[75] Cao H., Xu M., Li J. (2016). Effect of electro-acupuncture at zusanli point on the injury of the heart function in sepsis rats. *Medical Journal of Wuhan University*.

[76] Wu J., Lyu B., Gan T. (2017). Electroacupuncture improves acute bowel injury recovery in rat models. *Experimental and Therapeutic Medicine*.

[77] Chen Y., Lei Y., Mo L. (2017). Effect of electroacupuncture pretreatment with different waveforms in septic brain injury in rats. *Journal of Clinical Anesthesiology*.

[78] Wang C., Yao J., Shi X. (2018). Effects of electro-acupuncture on organ damage and inflammatory response in sepsis rats. *Clinical Journal of Medical Officers*.

[79] Zhang L., Huang Z., Shi X. (2018). Protective effect of electroacupuncture at zusanli on myocardial injury in septic rats. *Evidence-Based Complementary and Alternative Medicine*.

[80] Zhang Z., Shi Y., Cai D. (2018). Effect of electroacupuncture at ST36 on the intestinal mucosal mechanical barrier and expression of occludin in a rat model of sepsis. Acupuncture in medicine. *Journal of the British Medical Acupuncture Society*.

[81] Wu J., Wu W., Jiang R. (2014). Effect of electro-acupuncture at zusanli(ST36) on expression of Ghrelin and HMGB1 in the small intestine of sepsis rats. *Chinese Journal of Integrated Traditional Chinese and Western Medicine*.

[82] Xiao J., Zhang H., Chang J. L. (2016). Effects of electro-acupuncture at Tongli (HT 5) and Xuanzhong (GB 39) acupoints from functional magnetic resonance imaging evidence. *Chinese Journal of Integrative Medicine*.

[83] Yin C. S., Chae Y., Kang O. S. (2015). Deqi is double-faced: the acupuncture practitioner’s and the subject’s perspective. *Evidence-based Complementary and Alternative Medicine*.

[84] Smith-Edwards K. M., Najjar S. A., Edwards B. S. (2019). Extrinsic primary afferent neurons link visceral pain to colon motility through a spinal reflex in mice. *Gastroenterology*.

[85] Fang J. Q., Du J. Y., Fang J. F. (2018). Parameter-specific analgesic effects of electroacupuncture mediated by degree of regulation TRPV1 and P2X3 in inflammatory pain in rats. *Life Sciences*.

[86] Xiang X. H., Chen Y. M., Zhang J. M. (2014). Low- and high-frequency transcutaneous electrical acupoint stimulation induces different effects on cerebral mu-opioid receptor availability in rhesus monkeys. *Journal of Neuroscience Research*.

[87] Yu C. C., Wang Y., Shen F. (2018). High-frequency (50 Hz) electroacupuncture ameliorates cognitive impairment in rats with amyloid beta 1-42-induced Alzheimer’s disease. *Neural Regeneration Research*.

[88] Remick D. G., Newcomb D. E., Bolgos G. L. (2000). Comparison of the mortality and inflammatory response of two models of sepsis: lipopolysaccharide vs. cecal ligation and puncture. *Shock*.

[89] Buras J. A., Holzmann B., Sitkovsky M. (2005). Animal models of sepsis: setting the stage. *Nature Reviews Drug Discovery*.

[90] Dejager L., Pinheiro I., Dejonckheere E. (2011). Cecal ligation and puncture: the gold standard model for polymicrobial sepsis?. *Trends in Microbiology*.

[91] Hubbard W. J., Choudhry M., Schwacha M. G. (2005). Cecal ligation and puncture. *Shock*.

[92] Seemann S., Zohles F., Lupp A. (2017). Comprehensive comparison of three different animal models for systemic inflammation. *Journal of Biomedical Science*.

[93] Wichterman K. A., Baue A. E., Chaudry I. H. (1980). Sepsis and septic shock--a review of laboratory models and a proposal. *The Journal of Surgical Research*.

[94] Ruiz S., Vardon-Bounes F., Merlet-Dupuy V. (2016). Sepsis modeling in mice: ligation length is a major severity factor in cecal ligation and puncture. *Intensive Care Medicine Experimental*.

[95] Fink M. P. (2014). Animal models of sepsis. *Virulence*.

[96] Gonnert F. A., Recknagel P., Seidel M. (2011). Characteristics of clinical sepsis reflected in a reliable and reproducible rodent sepsis model. *The Journal of Surgical Research*.

[97] Bello S., Krogsboll L. T., Gruber J. (2014). Lack of blinding of outcome assessors in animal model experiments implies risk of observer bias. *Journal of Clinical Epidemiology*.

